# The Impact of a Mediterranean-like Diet with Controlled Protein Intake on the Onco-Nephrological Scenario: Time for a New Perspective

**DOI:** 10.3390/nu14235193

**Published:** 2022-12-06

**Authors:** Arianna Bettiga, Francesco Fiorio, Francesca Liguori, Federico Di Marco, Giulia Quattrini, Riccardo Vago, Domenico Giannese, Andrea Salonia, Francesco Montorsi, Francesco Trevisani

**Affiliations:** 1 Division of Experimental Oncology, Urological Research Institute, IRCCS Ospedale San Raffaele, 20132 Milano, Italy; 2 IRCCS San Raffaele Hospital, Direzione Sanitaria, 20132 Milano, Italy; 3 Emerging Bacterial Pathogens Unit, Division of Immunology, Transplantation and Infectious Diseases, IRCCS Ospedale San Raffaele, 20132 Milano, Italy; 4 Faculty of Medicine and Surgery, Università Vita-Salute San Raffaele, 20132 Milano, Italy; 5 Department of Clinical and Experimental Medicine, University of Pisa, 56121 Pisa, Italy

**Keywords:** nutrition, urological cancer, kidney health, onconephrology, CKD, malnutrition, moderately controlled protein diet, quality of life, multidisciplinary

## Abstract

Chronic kidney disease (CKD) represents a frequent comorbidity in cancer patients, especially for patients affected by urological cancers. Unfortunately, impaired kidney function may limit the choice of adequate oncological treatments for their potential nephrotoxicity or due to contraindications in case of a low glomerular filtration rate. For these patients, tailored nephrological and nutritional management is mandatory. The K-DIGO guidelines do not define whether the nutritional management of CKD could be useful also in CKD patients affected by urological cancer. In fact, in clinical practice, oncological patients often receive high-protein diets to avoid malnutrition. In our study, we investigated the nutritional and nephrological impact of a Mediterranean-like diet with a controlled protein intake (MCPD) on a cohort of 82 stage III-IV CKD patients. We compared two cohorts: one of 31 non-oncological CKD patients and the other of 51 oncological patients with CKD. The use of an MCPD had a favorable impact on both the oncological and non-oncological CKD patients with an amelioration in all the investigated parameters and with a better quality of life, with no cases of malnutrition or AKI.

## 1. Introduction

The global burden of cancer has been increasing steadily [1]. This is due to an aging population, improved diagnostic tools, novel therapies, (target therapies, checkpoint inhibitors or immune modulators) and a consequent decrease in mortality rate. In addition, nutritional care plays a central role in the management of cancer patients as a whole to ward off muscle wasting and malnutrition, a common complication among patients with cancer, that is associated with a higher rate of morbidity and mortality [2]. Cancer and renal disease can coexist and influence each other [3]. On one hand, the malignancy itself can directly cause kidney injury through primary lesions or metastasis or indirectly through immunologically induced nephrotoxicity. Furthermore, kidney injury among oncological patients can also be the result of side effects from chemotherapy or from surgical treatment. In particular, the incidence of nephrotoxic complications has increased with the use of new drugs and intensive or combined chemotherapy regimens [4] and, as a consequence, patients with underlying CKD have limited options for cancer therapies due to their decreased renal function. On the other hand, kidney function impairment can reduce the therapeutic options available for treatment and consequently the possibility of full recovery from the malignancy. Moreover, in the case of AKI, but more likely in the case of CKD, the kidney function impairment could increase the toxicity of the therapy and often requires treatment interruption.

It is well known that dietary interventions are essential to prevent and manage chronic conditions including cancer and renal diseases, and it is a useful tool for therapy when combined with a pharmacological approach as part of a multidisciplinary intervention. In the last few decades, the dietary approach for the reduction and management of the progression of CKD to end-stage renal disease (ESRD) has been a hot topic of research in the field of nephrology [5,6,7]. Despite there being some evidence-based studies suggesting that low-protein diets (LPDs) are able to compensate for the metabolic alterations resulting from kidney failure and have a possible role in delaying renal replacement therapy (RRT), they are still underutilized for several reasons with particular concern remaining over the risk of malnutrition [8,9,10,11,12]. A Mediterranean-like dietary regimen has all the features included in the guidelines for chronic disease prevention: an abundance of vegetables, grains and fibers, a controlled intake of animal products and processed meats with low sodium and a refined sugar intake. In this dietary regimen, the protein intake is also controlled and normalized (∼0.8 g/kg/die), which is useful for stage III and IV CKD patients. The quality of protein is one of the highlights: plant-centered diets are useful in maintaining phosphate serum levels and in decreasing net endogenous acid production thus keeping metabolic acidosis at bay [13,14]. Moreover, the Mediterranean diet has been shown to reduce inflammation and oxidative stress with positive effects on blood pressure, endothelial function, lipid and glycemic profile; all these effects may reduce the loss of renal function and allow for an improvement in the survival rate and quality of life of CKD patients [15,16,17,18].

Therefore, LPDs are associated with slower CKD progression if the protein intake is individualized [19]. Very low protein diets (VLPDs), which consist of a protein restriction of 0.3 g/kg/die, reduce the progression of stage IV-V CKD to ESRD, protecting residual renal function and preserving a good nutritional status [20,21,22]. However, if the reno-protective effects of a Mediterranean like-diet moderately restricted in protein (MRPD) in adults with advanced CKD (including transplant patients) has been well known, the use of the same diet to slow the onset of renal damage in onco-nephrological patients still remains controversial [23]. 

Onco-nephrology represents a challenge for both oncologists and nephrologists to manage the interaction between the kidneys and cancer, not only to avoid AKI due to chemotherapy and/or cancer, but also to uncover the most effective treatment and the best management for the patient with CKD and a concomitant neoplasm [24,25]. Therefore, the use of a tailored diet will play a pivotal role in daily clinical management in the future. ESPEN (European Society for Clinical Nutrition and Metabolism) guidelines for nutrition in cancer discourage patients affected by neoplasms from following a protein-restricted diet and suggest a higher daily protein intake (1.2–1.5 g/kg/day) to avoid the risk of malnutrition [23]. Furthermore, systemic inflammation in these patients alters the synthesis and metabolism of proteins, carbohydrates, and lipids; for these reasons, specific nutritional therapy combined with medical expertise and pharmaceutical interventions in a multidisciplinary approach may mitigate the negative impact of inflammation. Despite being impaired, protein synthesis is not completely halted and is therefore responsive to the dietary intake of amino acids, as long as adequate amounts are met [26,27,28]. While these positions reinforce the enthusiasm of teams using low-/normalized-protein diets for patients with CKD and cancer, it remains a therapy with its pros and cons. 

The aim of our study was to investigate the safety of an MCPD in CKD patients with cancer and test its efficacy in improving nutritional and nephrological status as well as malnutrition-related parameters, all while preserving a high quality of life.

## 2. Materials and Methods

A retrospective study was conducted in a cohort of 82 patients enrolled in a tertiary care research institute between 2018 and 2021: the control group (CT) was composed of 31 patients affected by CKD alone (GFR < 60 mL/min/1.73 m^2^, K-DIGO 2012 classification) and 51 patients with CKD and a recent history of urological malignancies (within 12 months) were part of the case group (CS). Each patient underwent an initial nephrological and nutritional evaluation and was subsequently subjected to an MCPD for a period of 6 months. Daily energy intake was 30–35 kcal/kg/day, and for this purpose, protein-free commercial foods were introduced if necessary. Any supplementation (vitamin D, calcium, folic acid, vitamin B12 or others) was administered to patients who required specific interventions to correct deficiencies, all according to the good clinical practice criteria. Laboratory tests such as serum creatinine, serum Cystatin C, serum Bicarbonate, serum Uric Acid, serum Vitamin D, serum Albumin, serum Potassium, serum Urea and measured GFR using Iohexol clearance tests were included at the study baseline and at the end of the study. Nutritional status was clinically recorded at each visit, taking into account the general clinical status, weight, blood pressure, the presence of edema, and bio-impedance, with particular attention to the decrease in body cellular mass from baseline.

**Figure 1:**
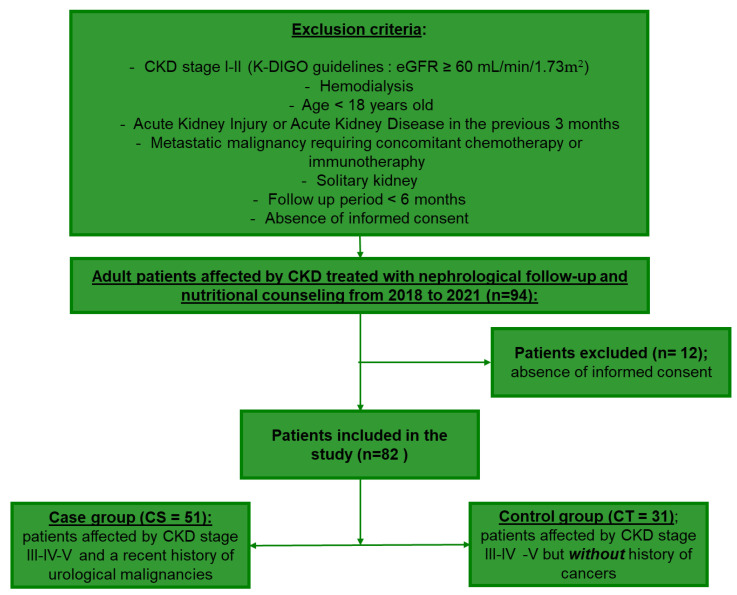
Flow chart for cohort assembly.

Figure 1 shows a flow chart outlining the inclusion and exclusion criteria for cohort assembly. Between 2018 and 2021, 94 CKD patients present in the internal database were included in the study. Exclusion criteria applied for cohort selection were: age < 18 years, eGFR ≥ 60 mL/min/1.73, renal function not stable for 3 months, end-stage renal disease requiring hemodialysis, solitary kidney, concomitant chemotherapy or immunotherapy, the presence of metastasis and the absence of informed consent. In total, 12 patients were excluded from the study for the absence of informed consent, and the remaining population was subsequently divided into the CS and CT groups.

We considered the following patient data: age, gender, body mass index (BMI), type of cancer (kidney, bladder, prostate, urothelial), hypertension, diabetes and medical therapy (ACE inhibitors (ACEi), angiotensin II receptor blockers (ARBs), calcium antagonists, beta-blockers and diuretics). 

The glomerular filtration rate (GFR) was estimated at each timepoint using the creatinine-based estimated glomerular filtration rate (eGFR) formulas: CKD-EPI (2012) [29]. The CKD categories were assigned according to the KDIGO guidelines [30]. The gold standard measurement using Iohexol plasma clearance was employed, to eliminate possible bias linked with sarcopenia for eGFR [31]. 

The study received the approval of the Institutional Ethical Committee (San Raffaele Hospital, Milan, Protocol Code/Acronym URBBAN, approval date 3 March 2014) and informed consent was obtained from all of the patients included in the study. All the experimental procedures involving human biological material were carried out in compliance with the approved guidelines and according to good clinical practice.

### 2.1. Assessment of Nutritional Status and Quality of Life

In order to assess the patients’ nutritional status, anthropometric measurements were performed: for each patient we collected body weight and height in order to calculate the corresponding Body Mass Index (BMI), Waist Circumference and Waist-Hips ratio. Bioelectrical Impedance Analysis (BIA) was used to study the patients’ body composition: we collected data for Phase Angle (PA), Body Cellular Mass (BCM)—Height^2^ ratio, Extracellular mass (ECM)—BCM ratio, Extracellular water (ECW)—Intracellular Water (ICW) ratio, Total Water (TBW%), Fat Mass (FM)—Height^2^ ratio and Free Fat Mass (FFM)—Height^2^ ratio and Mid-Upper Arm Muscle Circumference (MAMC). MAMC was calculated using the formula MAMC = Mid-arm Circumference − (3.1415 × Triceps Skinfold Thickness (TSF)) [32]. The Malnutrition Universal Screening Tool (MUST) was used to identify malnutrition. 

In order to evaluate the impact of the nutritional intervention on the perceived quality of life, a QoL-Short Form 36 (SF36) questionnaire was administered to patients. SF36 consisted of a total of 36 items assessing the impact on the following 8 domains: 1. Emotional Limitations; 2. Health Perceptions; 3. Mental Health; 4. Pain; 5. Physical Functioning; 6. Physical limitations; 7. Social Functioning; 8. Vitality.

Each of the summary scores was transformed into a linear scale ranging from 0 to 100 points, with higher scores indicating a better QoL; outcomes higher than 50 are considered positive. 

### 2.2. Statistical Analysis 

A comparison between numerical variables was performed using linear regressions; between groups using Kruskal–Wallis rank sum test (multiple groups) and Wilcoxon test (two groups) for numerical variables and Pearson’s Chi-square test for categorical variables. Significance level was considered for *p* < 0.05 using “holm” correction for multiple tests. Results are reported with median and interquartile range (q25–q75). Data analysis was performed using programming language R and RSTUDIO integrated development environment.

## 3. Results

### 3.1. Clinical and Demographic Findings of the Patients

Table 1 shows the descriptive statistical analysis of the cohort at the baseline, before any treatment was initiated. In total, 10 patients (12.2%) had diabetes and 62 patients (75.6%) had hypertension and, among the latter, 54 patients used anti-hypertensive drugs (ACEi, ARBs, calcium antagonists, beta-blockers and diuretics). Within the CT group, 22 were males and 9 were females who, all together, had a mean BMI of 25.1 (23.1–29.9), while within the CS group there were 41 males and 10 females, with a mean BMI of 26.1 (23.6–29.7). BMI classification shows that there were no underweight individuals among the patients involved in the study, 43.9% of the population were in a healthy weight range, 31.7% were overweight and 24.4% were obese. Table 1 also shows the patients who received supplementation to correct their vitamin D deficiency: 36.2% of the cohort received supplementation, specifically 51.7% of the CT group and 27.5% of the CS group.

Table 2 shows the tumor type and prevalence of each of these in the CS group of the cohort, as well as the surgery performed to cure them. Every one of the patients included in the study had no malignancy recurrence, no metastasis and stable follow-up conditions.

### 3.2. Overall Improvement in the Anthropometric Indices and Parameters following the Nephrologist Nutritionist Combined Approach (NNCA) in Both CS and CT Groups

By analyzing the variation for the entire population following the nephrologist-nutritionist combined approach (NNCA) intervention (Table 3), we observed an overall improvement in most of the anthropometric parameters considered. A small but significant increase in the PA indicated a good nutritional status in the population, where cellular health was preserved. We noticed, in fact, an increase in the BCM index and a stable but significant ECM/BCM ratio which indicated a healthy fluid balance and absence of malnutrition in the majority of the population. In terms of fluid balance, ECW/ICW ratio also remained significantly stable following the NNCA treatment, while TBW% followed an upwards trend which can be explained by a parallel increase in FFM. This increase in FFM was associated with a concomitant decrease in FM and this, overall, explained the significant reduction in the mean BMI of the population. There was also a reduction in the waist circumference which, together with a non-significant decrease in hip circumference, allowed the waist-hip ratio to remain very constant throughout the treatment period. The variation of the MAMC parameter did not show significance in this analysis.

### 3.3. NNCA Caused a Decrease in Malnourished Individuals over the Course of the Follow-Up Period in CS Patients but Not in the CT Group

Table 4 collects the percentages for malnourished individuals according to cut-offs found in the literature [33,34]. The analysis showed an improvement in malnutrition status only in the population that was part of the CS group following the NNCA, with the percentages of affected individuals dropping only in the concomitance of neoplasia. The malnourished patients within the CT group remained constant independently of the other variables. This proved that the MCPD was beneficial for onco-nephropathic patients despite the guidelines and the literature stating otherwise.

### 3.4. Circulating Levels of Urea and Vitamin D Are Positively Altered by NNCA in Both CS and CT Patients

Compared to the anthropometric parameters listed previously, the interpretation of the nephrological scenario is more complex and probably influenced by the follow-up duration which may have been too short to allow for evident amelioration. By observing Table 5, we can appreciate how only Urea and Vitamin D levels positively changed and reached statistical significance. In particular, Creatinine and Cystatin C levels remained stable; the variation in eGFR and mGFR did not reach statistical significance. 

When focusing on vitamin D plasmatic levels by dividing the cohort into supplemented and non-supplemented patients (Table 6), the Δ median of both groups of patients shows an increase in the circulating levels of vitamin D. A non-significant *p*-value explains that there is no difference in the observed changes in the levels of vitamin D between the two groups, hence, this analysis shows that the MCPD was able to boost vitamin D levels in the population independently from the supplementation.

### 3.5. The Stratification of the Outcomes Outlines That the Response of the CS and CT Groups Is Analogous

Table 7 shows how each group of the cohort responded to the NNCA by comparing parameters before and after the MCPD. *p*-values for all parameters were close to 1. These data are consistent with what has been previously described for Table 1, where we saw that the characteristics at the baseline were similar (as described by the non-significant *p*-values obtained and reported in the same table).

### 3.6. SF36 Questionnaires Outlined a Good Perceived Quality of Life in the Entire Cohort following NNCA

**Figure 2:**
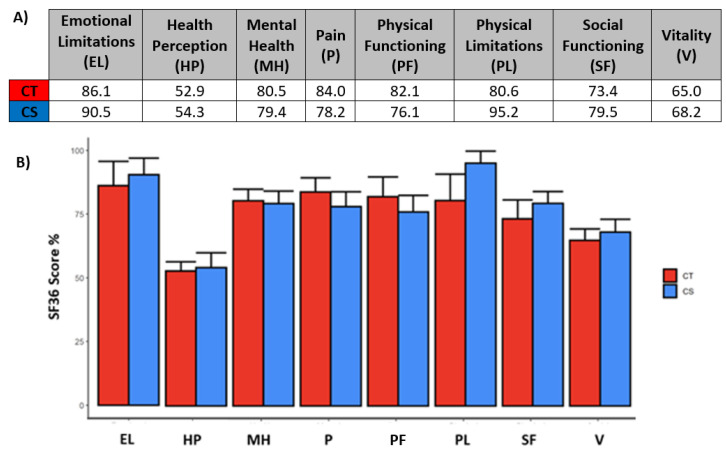
(**A**)—Numerical values for the 8 scales in the QoL SF36 shown in Figure 2B. (**B**)—Quality of life score evaluated through SF36 questionnaires for the 8 domains of the exam: 1. EL Emotional Limitations; 2. HP: Health Perceptions; 3. MH: Mental Health; 4. P: Pain; 5. PF: Physical Functioning; 6. PL: Physical Limitations; 7. SF: Social Functioning; 8. V: Vitality.

**Figure 3:**
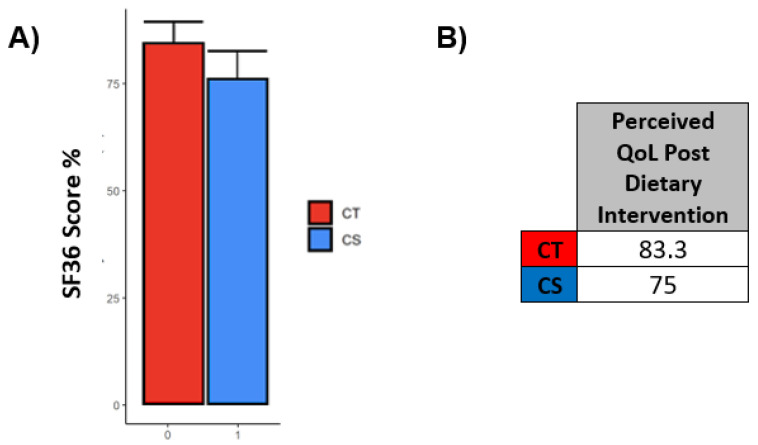
(**A**)—Perceived Quality of life scores after dietary intervention evaluated through SF36 questionnaire; (**B**)—Numerical values for the graphs shown in Figure 3A.

Figure 2A shows the results obtained for all eight domains of health through the evaluation of the results gathered using the QoL-SF36 questionnaire and Figure 2B shows the numerical values obtained for each scale assessed; similarly, Figure 3A,B display the same set of information regarding the QoL perceived by the patients of the cohort after the dietary treatment. The results outlined scores ranging between 52.9 and 95.2, highlighting a good perceived quality of life in the subjects treated with the combined approach. However, we can observe that health perception had the lowest scores among the eight assessed scales. Except for “Physical limitations”, all parameters followed a very similar trend between the two groups. The scores were all above 50, which indicated that the NNCA was effective in maintaining a good perceived quality of life overall but, by imposing limitations on their lifestyles, it affected their social activities, as well as increasing their perception of their pathological condition. Furthermore, all comparisons between groups were not statistically significant, indicating that the answers given by the two groups were analogous. Only one evaluation of the QoL was carried out, impeding the comparison of the outcomes before and after the treatment; for this reason, there was no real indication of a possible improvement or deterioration of the QoL as a consequence of the NNCA.

## 4. Discussion

The findings of our study provide evidence that the use of an MCPD in CKD-associated cancer patients is equally as effective as it would be in patients with non-oncological CKD. K-DIGO guidelines and nephrological clinical practice highlight that LPDs represent the gold standard of nutritional therapy in patients with stage III or higher CKD with the aim of slowing the progression to ESRD [35]. Our results showed that protein restriction, as well as energy balance and the reduction of phosphorus and salt intake, is the right therapy for this subset of patients.

Sixteen randomized, controlled trials show that the risk of progression to ESRD is significantly lower in those who received an LPD (0.6 to 0.8 g/kg/die) when compared with those who received higher-protein diets [36]. Among them, several meta-analyses underline that an LPD regimen is also associated with a lower incidence of metabolic acidosis, hyperazotemia, uremic symptoms, overt proteinuria and bone and mineral disorders [37,38,39,40,41,42,43]. As a results, different works clearly demonstrate that LPD is also able to delay the necessity of beginning renal replacement therapy [44].

However, when focusing on the impact a low-protein dietary regimen has on the supportive care of cancer patients, the relationship remains uncertain and not well elucidated, especially in onco-nephrological patients. ESPEN guidelines do not advise the application of such a diet for the management of cancer patients, who instead require a daily protein intake of 1 g/kg/die or more to avoid malnutrition and sarcopenia [45,46].

Our study highlights that the use of an MCPD in cancer patients with concomitant stage IIIa, IIIb and IV CKD improves the nephrological scenario as well as the nutritional status, without the risk of malnutrition. In particular, the data presented suggest that the MCPD was able to improve on several parameters for nutritional status in both groups, in particular PA and the ECM/BCM ratio, which underline the lack of malnutrition. The ECM/BCM ratio is often used to identify fluid imbalances and malnutrition and, in normal subjects, this value rages between 0.85 and 1.00 [47,48]. The median value for our patients remained significantly constant at 0.9 during the follow-up period and, furthermore, the number of patients we identified as malnourished based on the cut-off value of ECM/BCM ratio >1.2 decreased in the CS group following the NNCA. In the CT group, instead, the ECM/BCM ratio remained constant. This is in line with the results obtained for the other query used based on PA (for which the cut-off was <4.5°) where the outcome was equal to the previous. This result is of great significance since, as we have previously seen, the main contraindication with applying a non-high-protein diet to a population of oncological patients is the risk of malnutrition and sarcopenia; not only did these patients not develop these conditions, but the treatment was able to improve their nutritional status. Regarding the decreased sarcopenia risk, our population responded well to the treatment since they were able to overall lower their BMI while increasing their FFM and lowering their FM. While TBW% was also increased, the ECW/ICW ratio remained constant, showing that there was no induced edema as a response to the NNCA. Furthermore, the stable data of the MAMC parameter highlighted that muscular integrity was maintained throughout the follow-up period and these data can be supported by the small increase in the BCM index. Another interesting result is the increase in the BCM in the CS group. As mentioned, PA is usually decreased in sarcopenic patients [49]; our results show that the diet did not induce the lowering of PA, but was instead beneficial for oncological patients on a short-term observation. 

The impact that the NNCA had on the patients’ perceived QoL was overall positive since values for all the eight domains in the SF-36 questionnaire were increased to high scores and did not appear to be influenced by physical health and emotional status. Despite this, we observed that social activities were greatly and negatively affected. Harsh clinical monitoring and especially food choice limitations may have increased the perception of their pathological condition, thereby increasing the sense of responsibility in adhering to the nutritional therapy. All this, as a consequence, improved their physical health but, on the other hand, demotivated them and worsened their attitudes towards social habits (restaurant dining, travel, etc.). When introducing a new lifestyle into a patient’s routine, it is essential to take into consideration the impact that this has on their day-to-day life; adjusting a nutritional therapy to better suit their needs is the basis of a good personalized nutritional intervention and should be taken into consideration when dealing with patients. Our approach was well accepted and tolerated by our cohort of patients and compliance to the treatment was very high, suggesting it was effectively integrated into their normal routine. Despite this, it is safe to assume that there is always a possibility for improvement.

Regarding the nephrological data, our study highlighted that eGFR and mGFR values remained stable, with minor fluctuations over time and with no observed statistically significant changes. These results are not surprising and require two important considerations. First, patients with AKI were excluded from enrollment, as were those with other acute comorbidities, since their inclusion could have resulted in false baseline levels of serum creatinine or cystatin C. For this reason, we selected only patients with stable renal function for at least 3 months (following the K-DIGO guideline definition of CKD stages) in order to measure the real impact of a new dietary regimen and avoid the potential external bias due to medical therapies for other conditions such as AKI or AKD. The second consideration is the short period of observation. In fact, if 6 months represents an optimum timeframe for nutritional parameters variations, the period length does not allow us to define a worsening or an improvement in renal function in terms of GFR. Therefore, a rapid increase in eGFR or mGFR only thanks to an improvement of the patients’ dietary habits would be unlikely in these patient cohorts.

The main nephrological aspect that our study highlights is the decrease in urea levels in each study cohort due to the application of the NNCA. Azotemia is a biochemical abnormality, defined as an elevation or buildup of nitrogenous products. These products increase in CKD patients due to the loss of kidney function resulting in an inability to excrete uremic toxins [50]. As a consequence, several systemic side effects such as platelet dysfunction and bleeding, encephalopathy, peripheral neuropathy, nausea, vomiting, hypothermia and itching can irreversibly compromise the lifespan of CKD patients, with a high risk of mortality. Moreover, hyperazotemia represents one important nephrological situation that requires urgent hemodialysis [51,52]. Therefore, new therapeutic strategies to reduce the levels of plasmatic urea are required in daily clinical practice. Our study clearly underlined that an NNCA in onco-nephrological patients is able to significantly decrease urea levels in all patients in only 6 months, suggesting that this dietary regimen could help the management of these patients. Before our study, this approach was not intuitive, because oncological patients are often treated with high protein intakes in order to avoid malnutrition and catabolism, leading to an increase in blood urea. Finally, a significant increase in plasmatic levels of vitamin D was observed in the population which, as Table 6 shows, is apparently independent of supplementation and is not group-specific. Our results could indicate that an MCPD in the context of an NNCA is able to boost vitamin D plasmatic levels in both nephropathic and onco-nephropathic patients independently from vitamin D supplementation. Further investigations on this topic will have to take place in order to confirm this result. 

Our study has some important limitations.

The sample size was limited and, as a result, it was not possible to stratify the cohort, which would have been useful for examining the heterogeneity of the outcomes and how they could be affected by confounding variables. The biggest limitation of all concerning the cohort’s numerosity is the lack of a sample size analysis that would have been able to suggest the appropriate number of patients to enroll in order to maximize statistical power. 

The study was also limited by the observation period; only two visits were considered for each patient, approximately 6 months apart, which may not be enough to observe changes in the nephrological status of these patients. Furthermore, with a more intensive follow-up consisting of more time and more than two visits, it would be easier to explore the trend and speculate on what the effects over a longer period of time may be. 

Additionally, the design of the study was retrospective with all of the limitations associated with such studies.

Despite the existence of conflicting evidence in the literature which advises an LPD for CKD patients but discourages its use in cancer patients, we demonstrated for the first time that an MCPD is able to prevent the progression of pathological conditions in onco-nephrological patients and that this diet serves well for nephrological patients with or without neoplasms. One of the strengths of our study was the evaluation of parameters such as PA, FM%, FFMI, BCMI and ECW/ICW, which have already been validated and have demonstrated to be good nutritional status predictors both in nephropathic and oncologic patients. As a consequence, obtaining statistically significant outcomes for a great number of these variables outlines the efficacy of our treatment, which will have to be tested on a larger group of patients to confirm the trends observed.

## 5. Conclusions

In our study, we demonstrated for the first time that an MCPD in combination with medical intervention is safe, with no evidence of sarcopenia, and able to maintain the stability of the condition and reduce the uremic toxin value of patients affected by CKD and cancer, as well as in the patients with CKD alone. The synergistic management by nephrologists and nutritionists in our study highlights the value of a multidisciplinary approach over individual ones.

## Figures and Tables

**Table 1:** Descriptive statistical analysis; **^1^** shows the *p*-values obtained through Kruskal–Wallis Rank Sum Test; **^2^** shows the *p*-values obtained through Pearson’s Chi-Squared Test.

		N (% on Total)			*p*-Value
**Number of patients**		82	**Onco**	51 (62.2%)	/
			**Nephro**	31 (37.8%)	
**Age**		69.8 ± 10.3	**Onco**	71.6 (±8.8)	0.9 ^1^
			**Nephro**	66.5 (±12.1)	
**Gender**		♂ 63 (76.8%)	**Onco**	41 (65.1%)	0.327 ^2^
			**Nephro**	22 (34.9%)	
		♀ 19 (23.2%)	**Onco**	10 (52.6%)	
			**Nephro**	9 (47.4%)	
**BMI**		25.9	**Onco**	26.1 (23.6–29.7)	0.450 ^2^
			**Nephro**	25.1 (23.1–29.9)	
**BMI Classification**	**Underweight (<18.5)**	0	**Onco**	0	0.665 ^2^
			**Nephro**	0	
	**Healthy weight (18.5–24.9)**	36 (43.9%)	**Onco**	21 (58.3%)	
			**Nephro**	15 (41.7%)	
	**Overweight (25–29.9)**	26 (31.7%)	**Onco**	18 (69.2%)	
			**Nephro**	8 (30.8%)	
	**Obese (>30)**	20 (24.4%)	**Onco**	12 (60%)	
			**Nephro**	8 (40%)	
**CKD**	**Stage III a**	21 (25.9%)	**Onco**	15 (29.4%)	0.37 ^2^
			**Nephro**	6 (20%)	
	**Stage III b**	32 (39.5%)	**Onco**	22 (43.1%)	
			**Nephro**	10 (33.3%)	
	**Stage IV**	28 (34.6%)	**Onco**	14 (27.5%)	
			**Nephro**	14 (46.7%)	
**Diabetes**		10 (12.2%)	**Onco**	8 (15.7%)	0.153 ^2^
			**Nephro**	2 (6.5%)	
**Hypertension (HT)**		62 (75.6%)	**Onco**	37 (72.5%)	0.243 ^2^
			**Nephro**	25 (80.6%)	
**HT Therapy**	**ACEi**	13 (21%)	**Onco**	9 (69.2%)	0.63 ^2^
			**Nephro**	4 (30.2%)	
	**Beta-Blockers**	11 (17.7%)	**Onco**	9 (81.8%)	0.19 ^2^
			**Nephro**	2 (18.2%)	
	**ARBs**	15 (24.2%)	**Onco**	9 (60.0%)	1 ^2^
			**Nephro**	6 (40.0%)	
	**Calcium Antagonists**	20 (32.3%)	**Onco**	10 (50.0%)	0.43 ^2^
			**Nephro**	10 (50.0%)	
	**Diuretics**	16 (25.8%)	**Onco**	8 (50.0%)	0.53 ^2^
			**Nephro**	8 (50.0%)	
**Vitamin D**	**No Deficiency**	51 (63.8%)	**Onco**	37 (72.5%)	0.30 ^1^
			**Nephro**	14 (48.3%)	
	**Deficiency**	29 (36.2%)	**Onco**	14 (27.5%)	
			**Nephro**	15 (51.7%)	

**Table 2:** Occurrence of urological cancers in the CS cohort. The total of the neoplasms present in the population (n = 55) is bigger than the number of patients (n = 51) because some patients had two primitive tumors in different locations, not as a consequence of metastasis.

Tumor Location	Total	Surgery Performed	Frequency
**Kidney**	26	Radical Nephrectomy	16
		Partial Nephrectomy	10
**Prostate**	6	Prostatectomy	6
**Bladder**	17	Cistectomy	14
		TURBK	3
**Urothelium**	6	Nephroureterectomy	4
		TURBK	2

**Table 3:** Anthropometric indices and parameters at pre- and post-dietary and nephrological intervention (annotations follow the format: mean [confidence interval]). * indicates statistical significance for *p* ≤ 0.05, ** for *p* ≤ 0.01, *** for *p* ≤ 0.001.

	Before Dietary Intervention	After Dietary Intervention	*p*-Value
**Phase Angle (PA)**	5.7° [5.2;6.4]	5.8° [5.3;6.4]	0.0082 **
**ECM/BCM**	0.9 [0.8;1.0]	0.9 [0.8;1.0]	0.0049 **
**BCM/h^2^**	11 kg/h^2^ [9.5;12.2]	11.5 kg/h^2^ [9.8;12.7]	0.052
**BMI**	25.9 kg/h^2^ [23.5;29.8]	25.3 kg/h^2^ [23.3;28.3]	0.0015 **
**Waist Circumference**	95.8 cm [85.2;105.5]	93 cm [85.2;100]	0.00045 ***
**Waist-Hip Ratio**	0.9 [0.9;1]	0.9 [0.9;1]	0.012 *
**ECW/ICW**	0.9 [0.8;1]	0.9 [0.7;1]	0.00044 ***
**TBW (% on body weight)**	59.3 [54.4;61.8]	61.1 [56.2;63.6]	0.0005 ***
**MAMC**	25.9 mm [24.4;27.7]	25.5 mm [24;27.7]	0.17
**FM/h^2^**	5.2 kg/h^2^ [3.5;7.2]	3.8 kg/h^2^ [2.8;6.1]	0.000012 ***
**FFM/h^2^**	21 kg/h^2^ [19.6;22.4]	21.4 kg/h^2^ [19.8;23]	0.0002 ***

**Table 4:** Percentage of malnourished individuals in the two population groups. Cut-off values were taken from the literature: PA < 4.5° as a cut-off for protein-energy wasting in stage V non-dialysis CKD patients [33], ECM/BCM > 1.2 as an indicator of wasting and fluid overload in hemodialysis patients [34].

	Nephropathic		Onconephropathic	
	T0	T1	T0	T1
**PA < 4.5°**	12.9%	12.9%	3.9%	2%
**ECM/BCM > 1.2**	11.5%	11.5%	8.9%	4.4%

**Table 5:** Nephrological parameters before and after dietary and nephrological intervention (annotations follow the format: mean [confidence interval]). *p*-value was calculated with Kruskal–Wallis Sum Rank Test (*p* < 0.05). *** indicates statistical significance for *p* ≤ 0.001.

	Before Dietary Intervention	After Dietary Intervention	*p*-Value
**Creatinine**	1.8 mg/dL [1.5;2.4]	1.8 mg/dL [1.4;2.5]	0.94
**eGFR (epi 2012)**	37 mL/min/1.73m^2^ [23.6;46.2]	35.5 mL/min/1.73m^2^ [23;44.1]	0.67
**mGFR (Iohexol)**	37.5 mL/min/1.73m^2^ [32.4;44.1]	36.4 mL/min/1.73m^2^ [22.8;46.2]	0.34
**Urea**	68 mg/dL [47.2;92.8]	52 mg/dL [42;74.8]	0.0000033 ***
**Vitamin D**	25.5 ug/dL [18.5;37.4]	36 ug/dL [31.5;43]	0.000098 ***
**Bicarbonates**	24.4 mmol/L [22.8;27.2]	25.5 mmol/L [22.6;28]	0.95
**Cystatin C**	1.4 mg/L [1.2;1.8]	1.4 mg/L [1.2;1.9]	0.15
**Uric Acid**	5.5 mg/dL [4.8;6.9]	5.9 mg/dL [3.7;6.6]	0.52
**Potassium**	4.8 mmol/L [4.3;5.4]	4.7 mmol/L [4.4;5]	0.48

**Table 6:** Delta Median for vitamin D levels in the supplemented and the non-supplemented.

Vitamin D	ΔMedian	*p*-Value
**Supplemented**	5.9 [0.0;17.7]	0.78
**Not Supplemented**	11.0 [−1.2;12.3]	

**Table 7:** Comparison between the response of each group to the NNCA treatment (annotations follow the format: mean [confidence interval]).

	Nephropathic		Onconephropathic	
	**T0**	**T1**	**T0**	**T1**
**PA**	5.3° [4.9;6]	5.4° [5;6.2]	5.9° [5.4;6.6]	6° [5.6;6.5]
**BCM/h^2^**	11.1 kg/h^2^ [9;12.1]	11 kg/h^2^ [9.2;12.1]	11.1 kg/h^2^ [9.8;12.3]	11.6 kg/h^2^ [9.9;12.9]
**ECW/ICW**	0.9 [0.8;1]	0.9 [0.8;1]	0.8 [0.8;1]	0.8 [0.7;0.9]
**FFM/h^2^**	21.2 kg/h^2^ [19;23.2]	21.4 kg/h^2^ [19.2;23.3]	21 kg/h^2^ [19.8;22]	21.2 kg/h^2^ [20;23]
**eGFR (epi 2012)**	32.3 mL/min/1.73m^2^ [20.8;45]	36.7 mL/min/1.73m^2^ [21.6;42.6]	38.8 mL/min/1.73m^2^ [24.6;46.1]	34.5 mL/min/1.73m^2^ [23.8;46.7]
**mGFR (Iohexol)**	37.5 mL/min/1.73m^2^ [26.3;41.8]	38.3 mL/min/1.73m^2^ [21.9;45.3]	37.5 mL/min/1.73m^2^ [33;45]	36.4 mL/min/1.73m^2^ [23;46.2]
**Urea**	87 mg/dL [43;119.5]	58 mg/dL [42.5;79.5]	63 mg/dL [48;91]	51 mg/dL [42;69]
**Vitamin D**	32.2 ug/dL [21.6;42.6]	36.6 ug/dL [28.9;42.8]	24 ug/dL [15.7;35]	35.8 ug/dL [32;43]
**Bicarbonates**	23.6 mmol/L [22.5;24.3]	25 mmol/L [22.1;27.2]	25.8 mmol/L [24;28]	26.6 mmol/L [24.6;28.4]
**Uric Acid**	5.5 mg/dL [4.5;7.3]	4.6 mg/dL [3.6;6.5]	5.5 mg/dL [5.3;6.8]	6.2 mg/dL [4.5;6.6]

## Data Availability

All the data is stored in institutional repositories.

## References

[1] Kocarnik J.M., Compton K., Dean F.E., Fu W., Gaw B.L., Harvey J.D., Dhimal M. (2022). Cancer Incidence, Mortality, Years of Life Lost, Years Lived With Disability, and Disability-Adjusted Life Years for 29 Cancer Groups From 2010 to 2019: A Systematic Analysis for the Global Burden of Disease Study 2019. JAMA Oncol..

[2] Argiles J.M. (2005). Cancer-Associated malnutrition. Eur. J. Oncol. Nurs..

[3] Stengel B. (2010). Chronic kidney disease and cancer: A troubling connection. J. Nephrol..

[4] Malyszko J., Tesarova P., Capasso G., Capasso A. (2020). The link between kidney disease and cancer: Complications and treatment. Lancet.

[5] Rysz J., Franczyk B., Ciałkowska-Rysz A., Gluba-Brzózka A. (2017). The Effect of Diet on the Survival of Patients with Chronic Kidney Disease. Nutrients.

[6] Hanna R.M., Ghobry L., Wassef O., Rhee C.M., Kalantar-Zadeh K. (2020). A Practical Approach to Nutrition, Protein-Energy Wasting, Sarcopenia, and Cachexia in Patients with Chronic Kidney Disease. Blood Purif..

[7] Hsu H.J., Yen C.H., Wu I.W., Liu M.H., Cheng H.Y., Lin Y.T., Lee C.-C., Hsu K.-H., Sun C.-Y., Chen C.-Y. (2021). The association between low protein diet and body composition, muscle function, inflammation, and amino acid-based metabolic profile in chronic kidney disease stage 3–5 patients. Clin. Nutr. ESPEN.

[8] Kovesdy C.P., Kalantar-Zadeh K. (2016). Back to the future: Restricted protein intake for conservative management of CKD, triple goals of renoprotection, uremia mitigation, and nutritional health. Int. Urol. Nephrol..

[9] Ash S., Campbell K.L., Bogard J., Millichamp A. (2014). Nutrition Prescription to Achieve Positive Outcomes in Chronic Kidney Disease: A Systematic Review. Nutrients.

[10] Hahn D., Hodson E.M., Fouque D. (2018). Low protein diets for non-diabetic adults with chronic kidney disease. Cochrane Database Syst. Rev..

[11] Mitch W.E., Remuzzi G. (2016). Diets for patients with chronic kidney disease, should we reconsider?. BMC Nephrol..

[12] Obi Y., Qader H., Kovesdy C.P., Kalantar-Zadeh K. (2015). Latest consensus and update on protein-energy wasting in chronic kidney disease. Curr. Opin. Clin. Nutr. Metab. Care.

[13] Moorthi R.N., Armstrong C.L., Janda K., Ponsler-Sipes K., Asplin J.R., Moe S.M. (2014). The Effect of a Diet Containing 70% Protein from Plants on Mineral Metabolism and Musculoskeletal Health in Chronic Kidney Disease. Am. J. Nephrol..

[14] Moe S.M., Zidehsarai M.P., Chambers M.A., Jackman L.A., Radcliffe J.S., Trevino L.L., Donahue S.E., Asplin J.R. (2011). Vegetarian Compared with Meat Dietary Protein Source and Phosphorus Homeostasis in Chronic Kidney Disease. Clin. J. Am. Soc. Nephrol..

[15] Kelly J., Palmer S.C., Wai S.N., Ruospo M., Carrero J.-J., Campbell K., Strippoli G.F.M. (2016). Healthy Dietary Patterns and Risk of Mortality and ESRD in CKD: A Meta-Analysis of Cohort Studies. Clin. J. Am. Soc. Nephrol..

[16] Haring B., Selvin E., Liang M., Coresh J., Grams M.E., Petruski-Ivleva N., Steffen L.M., Rebholz C.M. (2017). Dietary Protein Sources and Risk for Incident Chronic Kidney Disease: Results From the Atherosclerosis Risk in Communities (ARIC) Study. J. Ren. Nutr..

[17] Lew Q.-L.J., Jafar T.H., Koh H.W.L., Jin A., Chow K.Y., Yuan J.-M., Koh W.-P. (2016). Red Meat Intake and Risk of ESRD. J. Am. Soc. Nephrol..

[18] Chen X., Wei G., Jalili T., Metos J., Giri A., Cho M.E., Boucher R., Greene T., Beddhu S. (2016). The Associations of Plant Protein Intake With All-Cause Mortality in CKD. Am. J. Kidney Dis..

[19] Metzger M., Yuan W.L., Haymann J.-P., Flamant M., Houillier P., Thervet E., Boffa J.-J., Vrtovsnik F., Froissart M., Bankir L. (2017). Association of a Low-Protein Diet With Slower Progression of CKD. Kidney Int. Rep..

[20] Fouque D., Laville M. (2006). Low protein diets for chronic kidney disease in non diabetic adults. Cochrane Database Syst. Rev..

[21] Caria S., Cupisti A., Sau G., Bolasco P. (2014). The incremental treatment of ESRD: A low-protein diet combined with weekly hemodialysis may be beneficial for selected patients. BMC Nephrol..

[22] Bellizzi V., Di Iorio B., De Nicola L., Minutolo R., Zamboli P., Trucillo P., Catapano F., Cristofano C., Scalfi L., Conte G. (2007). Very low protein diet supplemented with ketoanalogs improves blood pressure control in chronic kidney disease. Kidney Int..

[23] Arends J., Bachmann P., Baracos V., Barthelemy N., Bertz H., Bozzetti F., Fearon K., Hütterer E., Isenring E., Kaasa S. (2017). ESPEN guidelines on nutrition in cancer patients. Clin. Nutr..

[24] Fofi C., Festuccia F. (2021). Onconephrology: A New Challenge for the Nephrologist. Nephrol. Public Health Worldw..

[25] Kala J., Finkel K.W. (2021). Onconephrology. Crit. Care Clin..

[26] MacDonald A.J., Johns N., Stephens N., Greig C., Ross J.A., Small A.C., Husi H., Fearon K.C.H., Preston T. (2015). Habitual myofibrillar protein synthesis is normalin patients with upper GI cancer cachexia. Clin. Cancer Res..

[27] Winter A., MacAdams J., Chevalier S. (2012). Normal protein anabolic response to hyperaminoacidemia in insulin-resistant patients with lung cancer cachexia. Clin. Nutr..

[28] Baracos V.E. (2015). Skeletal muscle anabolism in patients with advanced cancer. Lancet Oncol..

[29] Levey A.S., Stevens L.A., Schmid C.H., Zhang Y.L., Castro A.F., Feldman H.I., Kusek J.W., Eggers P., Van Lente F., Greene T. (2009). A New Equation to Estimate Glomerular Filtration Rate. Ann. Intern. Med..

[30] Levin A.S., Bilous R.W., Coresh J. (2013). Chapter 1: Definition and classification of CKD. Kidney Int. Suppl..

[31] Gaspari F., Thakar S., Carrara F., Perna A., Trillini M., Aparicio M.C., Diadei O., Ferrari S., Cannatá A., Stucchi N. (2018). Safety of Iohexol Administration to Measure Glomerular Filtration Rate in Different Patient Populations: A 25-Year Experience. Nephron.

[32] Topolski T.D., LoGerfo J., Patrick D.L., Williams B., Walwick J., Patrick M.B. (2006). Peer Reviewed: The Rapid Assessment of Physical Activity (RAPA) Among Older Adults. Prev. Chronic Dis..

[33] Han B.-G., Lee J.Y., Kim J.-S., Yang J.-W. (2018). Clinical Significance of Phase Angle in Non-Dialysis CKD Stage 5 and Peritoneal Dialysis Patients. Nutrients.

[34] Ruperto M., Sánchez-Muniz F.J., Barril G. (2020). Extracellular mass to body cell mass ratio as a potential index of wasting and fluid overload in hemodialysis patients. A case-control study. Clin. Nutr..

[35] Bellizzi V., Cupisti A., Locatelli F., Bolasco P., Brunori G., Cancarini G., Caria S., De Nicola L., Conservative Treatment of CKD, Study Group of the Italian Society of Nephrology (2016). Low-Protein diets for chronic kidney disease patients: The Italian experience. BMC Nephrol..

[36] Aparicio M., Bellizzi V., Chauveau P., Cupisti A., Ecder T., Fouque D., Garneata L., Lin S., Mitch W., Teplan V. (2013). Do Ketoanalogues Still Have a Role in Delaying Dialysis Initiation in CKD Predialysis Patients?. Semin. Dial..

[37] Rhee C.M., Ahmadi S.-F., Kovesdy C.P., Kalantar-Zadeh K. (2017). Low-Protein diet for conservative management of chronic kidney disease: A systematic review and meta-analysis of controlled trials. J. Cachex- Sarcopenia Muscle.

[38] Kovesdy C.P., Anderson J.E., Kalantar-Zadeh K. (2008). Association of serum bicarbonate levels with mortality in patients with non-dialysis-dependent CKD. Nephrol. Dial. Transplant..

[39] Yan B., Su X., Xu B., Qiao X., Wang L. (2018). Effect of diet protein restriction on progression of chronic kidney disease: A systematic review and meta-analysis. PLoS ONE.

[40] Mitch W.E., Remuzzi G. (2004). Diets for patients with chronic kidney disease, still worth prescribing. J. Am. Soc. Nephrol..

[41] Fouque D., Aparicio M. (2007). Eleven reasons to control the protein intake of patients with chronic kidney disease. Nat. Clin. Pract. Nephrol..

[42] Cupisti A., D’Alessandro C., Morelli E., Rizza G.M., Galetta F., Franzoni F., Barsotti G. (2004). Nutritional status and dietary manipulation in predialysis chronic renal failure patients. J. Ren. Nutr..

[43] Fouque D., Wang P., Laville M., Boissel J.P. (2000). Low protein diets for chronic renal failure in non diabetic adults. Cochrane Database Syst. Rev..

[44] Verzola D., Picciotto D., Saio M., Aimasso F., Bruzzone F., Sukkar S.G., Massarino F., Esposito P., Viazzi F., Garibotto G. (2020). Low Protein Diets and Plant-Based Low Protein Diets: Do They Meet Protein Requirements of Patients with Chronic Kidney Disease?. Nutrients.

[45] Meza-Valderrama D., Marco E., Dávalos-Yerovi V., Muns M., Tejero-Sánchez M., Duarte E., Sánchez-Rodríguez D. (2021). Sarcopenia, Malnutrition, and Cachexia: Adapting Definitions and Terminology of Nutritional Disorders in Older People with Cancer. Nutrients.

[46] Bossi P., Delrio P., Mascheroni A., Zanetti M. (2021). The Spectrum of Malnutrition/Cachexia/Sarcopenia in Oncology According to Different Cancer Types and Settings: A Narrative Review. Nutrients.

[47] Talluri T., Lietdke R.J., Evangelisti A., Talluri J., Maggia G. (1999). Fat-Free mass qualitative assessment with bioelectric impedance analysis (BIA). Ann. New York Acad. Sci..

[48] Noce A., Vidiri M.F., Marrone G., Moriconi E., Bocedi A., Capria A., Rovella V., Ricci G., De Lorenzo A., Di Daniele N. (2016). Is low-protein diet a possible risk factor of malnutrition in chronic kidney disease patients?. Cell Death Discov..

[49] Di Vincenzo O., Marra M., Di Gregorio A., Pasanisi F., Scalfi L. (2020). Bioelectrical impedance analysis (BIA) -derived phase angle in sarcopenia: A systematic review. Clin. Nutr..

[50] Mehta R.L., Cerdá J., Burdmann E.A., Tonelli M., García-García G., Jha V., Susantitaphong P., Rocco M., Vanholder R., Sever M.S. (2015). International Society of Nephrology’s 0by25 initiative for acute kidney injury (zero preventable deaths by 2025): A human rights case for nephrology. Lancet.

[51] Yu M.K., Kamal F., Chertow G.M. (2019). Updates in Management and Timing of Dialysis in Acute Kidney Injury. J. Hosp. Med..

[52] Sawhney S., Fraser S.D. (2017). Epidemiology of AKI: Utilizing Large Databases to Determine the Burden of AKI. Adv. Chronic Kidney Dis..

